# GRK5 is required for adipocyte differentiation through ERK activation

**DOI:** 10.1038/s41366-025-01712-w

**Published:** 2025-01-21

**Authors:** Mary E. Seramur, Bailey McDonald, Matt Davis, Tony E. Reeves, Leah C. Solberg Woods, Chia-Chi Chuang Key

**Affiliations:** 1 Department of Internal Medicine, Section on Molecular Medicine, Wake Forest University School of Medicine, Winston Salem, NC 27101 USA; 2 Wake Forest Institute for Regenerative Medicine, Wake Forest University School of Medicine, Winston Salem, NC 27101 USA

**Keywords:** Obesity, Fats

## Abstract

Previous studies have identified G protein-coupled receptor (GPCR) kinase 5 (GRK5) as a genetic factor contributing to obesity pathogenesis, but the underlying mechanism remains unclear. We demonstrate here that Grk5 mRNA is more abundant in stromal vascular fractions of mouse white adipose tissue, the fraction that contains adipose progenitor cells, or committed preadipocytes, than in adipocyte fractions. Thus, we generated a GRK5 knockout (KO) 3T3-L1 preadipocyte to further investigate the mechanistic role of GRK5 in regulating adipocyte differentiation. During adipogenic stimulation, GRK5 KO preadipocytes had decreased lipid accumulation and delayed mature adipocyte development compared to wildtype cells coupled with suppressed adipogenic and lipogenic gene expression. Beside GPCR signaling, RNA sequencing and pathway analysis identified insulin-like growth factor 1 (IGF-1) signaling to be one of the top 5 significantly dysregulated pathways in GRK5 KO cells. GRK5 KO cells also displayed decreased insulin-stimulated ERK phosphorylation, a downstream target of insulin-stimulated IGF-1 receptor activation, suggesting that GRK5 acts through this critical pathway to impact 3T3-L1 adipocyte differentiation. To find a more translational approach, we identified a new small molecule GRK5 inhibitor that was able to reduce 3T3-L1 adipogenesis. These data suggest that GRK5 is required for adipocyte differentiation through IGF-1 receptor/ERK activation and may be a promising translational target for obesity.

## Introduction

Prevalence of obesity continues to increase, along with related comorbidities [1–3]. Adipose tissue plays a central role in obesity resulting in adipocyte hyperplasia (increased adipocyte number) and/or adipocyte hypertrophy (increased adipocyte size), although the underlying causes of adipose tissue expansion are generally unknown. Our previous work identified G protein-coupled receptor (GPCR) kinase 5 (GRK5) as a candidate causal gene for visceral adiposity in outbred rats. Specifically, we identified a highly significant quantitative trait locus for visceral adiposity on rat chromosome 1. Although 12 genes fell in this region, only *Grk5* was identified as a full mediator of retroperitoneal and epididymal white fat mass using mediation analysis [4]. We further demonstrated that *Grk5* knock-down in 3T3-L1 preadipocytes led to decreased total triacylglycerol (TAG) accumulation in mature adipocytes [4]. That *Grk5* may be a causal gene for adiposity is supported by previous studies using a global GRK5 knockout (KO) mouse model where GRK5 KO mice exhibited protection against diet-induced increases in weight gain and fat mass compared to wildtype (WT) control mice, which may be due, in part, to decreased adipogenesis [5]. In addition to rodent studies, human GRK5 gene expression in subcutaneous white adipose tissue positively correlates with BMI (kg/m^2^) in the African American Genetics of Metabolism and Expression (AAGMEx) cohort (r = 0.22, *p* = 0.0003), a group of 256 African Americans with in depth glucometabolic phenotyping and adipose tissue transcriptome analysis [6]. These findings indicate that GRK5 is likely a causal gene for adiposity and may serve as a target to treat obesity. However, the mechanisms by which GRK5 contribute to adiposity and obesity remain unclear.

The GRK family consists of 7 proteins (GRK1-7) that can terminate GPCR signaling through phosphorylation of cognate receptors, resulting in recruitment of β-arrestin, which then facilitates receptor internalization and degradation [7]. The GTEx RNA sequencing dataset showed that subcutaneous white adipose tissue demonstrates the second highest GRK5 mRNA expression, with only lung being higher, in humans [8]. In mice, GRK5 mRNA tissue distribution is similar to that of humans (i.e., highest in the lung, followed by subcutaneous white fat) [9]. Although GRK5 is highly expressed in adipose tissue [8, 9], the Human Protein Atlas reported that GRK5 is present in endothelial cells and adipose progenitor cells (committed preadipocytes), but not in mature adipocytes, of human white visceral and subcutaneous fat pads [10]. This is in agreement with a recent single cell/nuclei RNA sequencing analysis of human and mouse white adipose tissue that revealed virtually no GRK5 transcripts in mature adipocytes [11, 12]. Thus, we have created a novel GRK5 KO 3T3-L1 preadipocyte cell line to investigate the role of GRK5 in the regulation of adipocyte differentiation and function.

3T3-L1 preadipocytes are differentiated into mature insulin-responsive adipocytes by exposing a quiescent population of confluent cells to a classical adipogenic cocktail, including 3-isobutyl-1-methylxanthine (IBMX), dexamethasone (DEX), and insulin, that activates a cascade of transcription factors. IBMX is used to increase cAMP levels to activate cAMP responsive-element binding protein (CREB) [13]. When activated, CREB translocates to the nucleus and binds to CRE to promote expression of CCAAT/enhancer-binding protein (C/EBP) and peroxisome proliferator-activated receptor gamma (PPARγ) [14, 15]. DEX is used to directly induce C/EBP and PPARγ activity [16, 17]. C/EBP and PPARγ coordinate the expression of numerous genes such as fatty acid synthase (FASN) for de novo fatty acid synthesis, acyl-CoA: diacylglycerol acyltransferase (DGAT) for fatty acid esterification, and cluster of differentiation 36 (CD36) for fatty acid transport, all of which are associated with the mature adipocyte phenotype.

Insulin, acting through the insulin-like growth factor-1 (IGF-1) receptor, a receptor tyrosine kinase, is also required to ensure complete conversion of 3T3-L1 preadipocytes into mature adipocytes [18]. Recently, the Farmer group demonstrated that insulin induces a robust transient activation of the extracellular signal-regulated kinase (ERK) pathway during the first 12 h of 3T3-L1 adipogenesis, and that this is required for subsequent adipocyte differentiation by activating C/EBP and PPARγ [19]. Insulin/IGF-1 receptor and ERK can communicate in several ways to activate adipocyte differentiation. In this study, we will explore a novel hypothesis that GRK5 is involved in adipocyte differentiation by regulating the insulin/IGF-1 receptor/ERK pathway suggesting that GRK5 is not only a GPCR kinase, but also governs receptor tyrosine kinase signaling in preadipocytes to control adipogenesis. These in vitro studies provide important mechanistic support for the role of GRK5 in adipogenesis and are in-line with previous in vivo studies which implicate GRK5 as an important obesity gene. Future studies are needed to better understand the role of this gene in vivo and to determine if it may be a useful target to treat obesity.

## Materials and methods

### Mice

Mice were housed in standard cages under a 12-h light cycle and 12-h dark cycle (dark from 6:00 PM to 6:00 AM) at standard ambient temperature and humidity conditions and were provided with ad libitum water and a standard chow diet (Purina-LabDiet, Prolab RMH 3000). For the mouse studies, a minimum of 5 mice per experimental group was calculated using G*Power software. This was based on the ability to detect with 80% power a 20% difference in whole body and fat pad weight with a SD of 10% based on a 2-sample t-test with alpha = 0.05 (2-sided). Mice were assigned randomly to different types of diets without being screened for inclusion or exclusion by the investigators.

Ethics approval: All experiments were performed using a protocol approved by the Institutional Animal Care and Use Committee at Wake Forest University School of Medicine in facilities approved by the American Association for Accreditation of Laboratory Animal Care.

To assess the impact of high fat diet on *Grk5* expression, 8-week-old male C57BL/6J mice (Jackson Lab, Bar Harbor, MN, USA, Strain #000664) were fed chow or a high fat diet (Envigo, Indianapolis, IN, USA, #TD 88137, 42% from fat, 0.2% total cholesterol or Research Diets Inc, New Brunswick, NJ, USA, #D12451, 45% from fat) for 16 weeks. Six-week-old female C57BL/6J mice were fed chow or a high fat diet (Research Diets Inc, New Brunswick, NJ, USA, #D12492, 60% from fat) for 12 weeks. Mice were fasted overnight before being euthanized, and adipose tissue (including visceral, subcutaneous, and brown) were collected and stored at −80 °C until used for gene or protein expression. To determine where in adipose tissue *Grk5* is expressed, 6-week-old male C57BL/6J mice were fed chow or a high-fat diet (Research Diets Inc, New Brunswick, NJ, USA, #D12492, 60% from fat) for 12 weeks. After 10 weeks on diet, mice went through EchoMRI™ analysis, which measures body composition (e.g., fat mass) of live mice. After 12 weeks on diet, mice were fasted for 16 h and epidydimal visceral white fat pads were harvested and used for adipose tissue digestion as described in the section below.

### Adipose tissue digestion

Briefly, adipose tissue was enzymatically digested in a digestion buffer (0.5 g of fat in 10 ml) containing 0.8 mg/ml of collagenase II (Worthington Biochemical Corp., Lakewood, NJ, USA), 3% of fatty acid free-BSA (Sigma-Aldrich, Burlington, MA, USA), 1.2 mM of calcium chloride (Sigma-Aldrich), 1 mM of magnesium chloride (Sigma-Aldrich), and 0.8 mM of zinc Chloride (Sigma-Aldrich) in Hanks Buffered Salt Solution (Life Technologies, Carlsbad, CA, USA) for 60 min in a shaking water bath at 37 °C with 200 rpm agitation. The fat digest was then filtered through a 250-um filter (Fisher Scientific, Pittsburgh, PA, USA). The adipocyte fraction and stromal vascular (SV) fraction were collected by centrifugation at 800 × *g* for 10 min. Red blood cells in the SV fraction were lysed using ACK lysis buffer. The adipocyte and SV fractions were treated with QIAzol Lysis Reagent (Qiagen, Venlo, Netherlands) and stored at −80 °C until used for gene expression.

### Cell cultures

The 3T3-L1 preadipocyte cell line was purchased from ATCC (CL-173™). The GRK5 KO 3T3-L1 preadipocyte was generated using CRISPR gene editing and provided by Synthego Corporation, Redwood City, CA (cells are available upon request). The guide sequence (i.e., TATGTGACAAGCAACCAATT) was designed to target exon 3 of *Grk5*. The KO clone was cut and had a nucleotide removed (i.e., A) during the non-homologous end joining repair process, resulting in a frameshift mutation that causes premature termination of translation at a new nonsense codon, as confirmed by the Sanger sequence (Supplementary Fig. 2A).

GRK5 KO 3T3-L1 and its wildtype (WT) control 3T3-L1 preadipocytes were first used to assess their proliferation rate using Click-iT® EdU cell proliferation kit (ThermoFisher Scientific, Waltham, MA, USA) based on the manufacture’s procedure. Briefly, cells were seeded into a 6-well plate with a density of 0.1 × 10^6^ per well in Dulbecco’s Modified Eagle Medium (DMEM, Gibco, Billings, MT, USA) supplemented with 10% iron-fortified calf serum (CS, Sigma-Aldrich) and 1% penicillin/streptomycin (P/S, Gibco) for 24 h. Cells were then treated with EdU (5-ethynyl-2’-deoxyuridine) solution and incubated for 24 h. EdU, a nucleoside analog of thymidine, was incorporated into newly synthesized DNA and fluorescently labeled with a bright, photostable Alexa Fluor™ 647 dye. Total DNA was stained using Hoechst 3342 and imaged using BioRad ZOE Fluorescent Cell Imaging System.

GRK5 KO 3T3-L1 and WT control 3T3-L1 preadipocytes were cultured and differentiated into adipocytes as described previously [20]. Briefly, preadipocytes were seeded at 0.05 × 10^6^ cells per well in a 6-well culture plate. Cells were cultured in DMEM supplemented with 10% iron-fortified CS and 1% P/S for 48 h until ∼90% cell confluence. Adipogenesis (Day 0) was induced by changing the medium to DMEM containing 10% fetal bovine serum (FBS, Sigma-Aldrich) plus an adipogenic cocktail (Sigma-Aldrich) including 1 μg/ml of insulin, 0.25 μM of dexamethasone, 0.5 mM of 3-isobutyl-1-methylxanthine, and 2 μM of rosiglitazone for 3 days (Day 3). Cells were then treated with 1 μg/ml of insulin only for 3 days (Day 6) and then without any adipogenic reagents for the next 3 days (Day 9). The medium was changed every 2 days. At Days 0, 3, 6 and 9 of differentiation, cells were stained with Oil-Red O and imaged using the BioTek Cytation C10 Confocal Imaging Reader (Agilent BioTek, Winooski, VT, USA) as well as lipid extracted for triacylglycerol (TAG) measurement as previously described [20].

In order to examine ERK expression, Day 2 differentiated WT and GRK5 KO cell cultures were serum starved overnight and then treated with 1 μg/ml of insulin for 5, 10, and 15 min. The cellular proteins were harvested as described in the section below for Western blot analysis.

### RNA extraction and real-time PCR

Total RNA was harvested from cells and tissues using QIAzol Lysis Reagent and isolated by following the protocol described in the RNeasy Lipid Tissue Mini Kit (Qiagen). The concentration and quality of RNA were determined using a Nanodrop (ThermoFisher Scientific) and standardized to 1 μg of RNA for cDNA synthesis. The cDNA was prepared with the OmniScript RT Kit (Qiagen) and stored at −20 °C until used for real-time PCR. Real-time PCR was performed in duplicate on the QuantStudio™ 3 systems (ThermoFisher Scientific) using TaqMan® Fast Advanced Master Mix and TaqMan® gene expression assays (ThermoFisher Scientific) including *Grk5* (Mm00517039_m1), *Cd36* (Mm00432403_m1), *Fabp4* (Mm00445878_m1), *Pparγ* (Mm0040940_m1), *Acc1* (Mm01304257_m1), *Fasn* (Mm00662319_m1), *Dgat2* (Mm00499536_m1), *Lipin1* (Mm00550511_m1), and *Lipin2* (Mm00522390_m1) with 18S rRNA (REF 4352655) as a housekeeping gene. Gene expression was normalized to the endogenous control gene 18S rRNA (REF 4352655) and analyzed using the 2ddCt method with 95% confidence.

### Protein extraction and Western blot

Total cellular protein was harvested in Pierce™ IP lysis buffer (ThermoFisher Scientific) supplemented with cOmplete™ EDTA-free Protease (Sigma-Aldrich) and PhosSTOP™ Phosphatase (Sigma-Aldrich) inhibitor tablets and frozen at −20 °C until used. Protein samples were normalized to 1 mg of protein, prepared in non-reducing laemmli buffer and DTT, and heated at 95 °C for 10 min. Protein was loaded and separated on a 4–20% polyacrylamide gel (Bio-Rad Laboratories, Hercules, CA, USA) and transferred to a 0.2 μm nitrocellulose membrane (Bio-Rad). Membranes were blocked in 5% non-fat milk in 1X Tris-buffered saline plus 0.1% Tween (TBST, Bio-Rad) for 2 h at room temperature. Primary antibodies were diluted in TBST with 1% non-fat dry milk and incubated overnight at 4 °C with gentle rocking. Primary antibodies were diluted as follows: GRK5 (#sc-518005, Santa Cruz Biotechnology, Santa Cruz, CA, USA) at 1:1000, GRK2 (#74761, ThermoFisher Scientific) at 1:1000, GAPDH (#sc-137179, Santa Cruz Biotechnology) at 1:1000, PPARγ (#2435, Cell Signaling Technology, Danvers, MA, USA) at 1:1000, CD36 (#28109, Cell Signaling Technology) at 1:1000, α-tubulin (#2144, Cell Signaling Technology) at 1:1000, Phospho-p44/42 ERK1/2 (#4376, Cell Signaling Technology) at 1:1000 and Total p44/42 ERK1/2 (#4695, Cell Signaling Technology) at 1:1000. Following overnight incubation, membranes were washed 3 times in TBST for 5 min with agitation and incubated with secondary antibody in 5% non-fat milk for 1 h at room temperature (ThermoFisher Scientific mouse and rabbit secondaries, 1:5000) with gentle rocking. Membranes were washed 3 times in TBST for 5 min with agitation. SuperSignal™ West Pico PLUS Chemiluminescent Substrate (ThermoFisher Scientific) was added to the membrane prior to imaging using the ChemiDoc Gel Imaging System (Bio-Rad). Protein expression was quantified using Bio-Rad ImageLab software.

### RNA sequencing and pathway analysis

GRK5 KO and WT cells were seeded and proliferated for 48 h. After 48 h, cells were treated with the adipogenic cocktail as described above for 6 h, and RNA was collected and extracted as previously described [20]. Total RNA was used to prepare cDNA libraries using the Illumina® TruSeq Stranded Total RNA with Ribo-Zero Gold Preparation kit (Illumina Inc., San Diego, CA, USA). The libraries were pooled and sequenced to an estimated target read depth of 40 M single-end 100 bp reads per sample on the Illumina NovaSeq 6000. For all samples, 80% of sequences achieved >Q30 Phred quality scores (FASTQC analysis, Babraham Bioinformatics). Adapter contamination was cleaned with Trimmomatic [21]. Reads were aligned to the murine reference genome mm39 using the STAR sequence aligner [22], and gene counts determined using featureCounts software [23]. Differentially expressed genes were identified using limma [24]. We then used Ingenuity Pathway Analysis (IPA) to identify top up-and down-regulated pathways.

### Computational molecular modeling and in vitro inhibition assays of a GRK5 inhibitor

A pyridine-based bicyclic compound of small molecule GRK5 inhibitor, GRK5-IN-2, was purchased from MedChemExpress (HY-136561). The binding affinity of GRK5-IN-2 to GRK5 and GRK2 were calculated using AutoDock Vina and visualized with PyMOL [25, 26]. The three-dimensional structures of human GRK5 (PDB 4TND) and GRK2 (PDB 5UUU) were downloaded from RCSB Protein Data Bank (PDB) and prepared using AutoDock Tools by removing all water and co-crystallized ligands, addition of polar hydrogens and assigning Kollman charges. Inhibitor isomeric simplified molecular input line entry system (SMILES) was obtained from PubChem and 3D coordinates generated using OpenBabel with the addition of hydrogens at pH 7.3 and partial charges calculated with a Gasteiger model.

The half maximal inhibitory concentration (IC50) of GRK5-IN-2 was determined using the ADP-Glo Kinase Assay (Promega, Madison, WI, USA) according to the manufacturer’s instructions. Briefly, a twofold serial dilution of GRK5-IN-2 was carried out in DMSO, and inhibitors were subsequently diluted into assay buffer to the final required concentrations. Each inhibitor dilution was transferred into a white 96-shallow well plate. GRK5 protein (final concentration at 0.5 mg/mL), ATP (final concentration at 25 µM), and casein (final concentration at 20 mg/mL) as the substrate were added to each well. Reactions were incubated for 120 min at room temperature. Then, ADP-Glo™ Reagent was added to each well and incubated at room temperature for 40 min to stop the kinase reaction and deplete the unconsumed ATP, leaving only ADP and a very low background of ATP. Kinase Detection Reagent was added and incubated at room temperature for 60 min to convert ADP to ATP and to introduce luciferase and luciferin to detect ATP using a plate-reading luminometer.

### Adipogenesis, fatty acid uptake, and lipogenesis

To assess the effects of GRK5-IN-2 on adipogenesis, WT 3T3-L1 preadipocytes were differentiated as described in the section above (Cell cultures), and concurrently treated without (DMSO vehicle) or with GRK5-IN-2 (20, 40, and 80 μM) for 7 days. Fatty acid uptake and incorporation into lipids as well as de novo lipogenesis were determined using [^3^H]-oleic acid and [^14^C]-acetic acid, respectively, following the procedure adapted from our previous study [20]. Day 3 differentiated WT 3T3-L1 cell cultures were pretreated without (DMSO vehicle) and with GRK5-IN-2 (40 μM) for 30 min, and then labeled with 0.5 μCi of [1,2-^14^C]-acetic acid (PerkinElmer, Waltham, MA, USA) or 5 μCi of [9,10-^3^H(N)]-oleic acid (PerkinElmer) plus 0.04 mM oleic acid (Sigma-Aldrich) conjugated with 0.01 mM fatty acid free-bovine serum albumin (BSA) of DMEM supplemented with 10% FBS, 1% P/S and 1 μg/ml of insulin for 0 (no radioisotopes), 30, 60 and 120 min. Following radiolabeling, cells were washed with ice-cold DPBS twice and lipid-extracted with hexane:isopropanol (3:2, vol:vol). Lipid classes from standards and cellular lipid extracts were separated by thin layer chromatography using Silica Gel plates and a solvent system containing hexane:diethyl ether:acetic acid (80:20:2, vol:vol:vol). Lipids were visualized by exposure to iodine vapor, and bands corresponding to TAG, free cholesterol (FC), cholesteryl ester (CE), and phospholipid (PL) were scraped and counted using a scintillation counter. After lipid extraction, cell residue was dissolved with 0.1 N of NaOH, and protein concentrations were measured using a Pierce™ BCA Protein Assay Kit for protein normalization of data.

### Statistics

Data are presented as mean ± standard error of the mean (SEM). All data points reflect biological replicates. Binary comparisons are performed using two-tailed Student’s *t* test. Datasets comparing the effect of a single independent variable on more than two groups are assessed by one-way ANOVA followed by Dunnett’s correction. Datasets containing groups defined by two independent variables (genotype, time) are assessed by two-way ANOVA with Sidak’s correction. Prism 10 software (GraphPad) is used to perform statistical analyses (Statistical significance *p* < 0.05) and generate graphical representations of data.

## Results

### *Grk5* is highly expressed in the stromal vascular fraction of mouse white adipose tissue

**Fig. 1:**
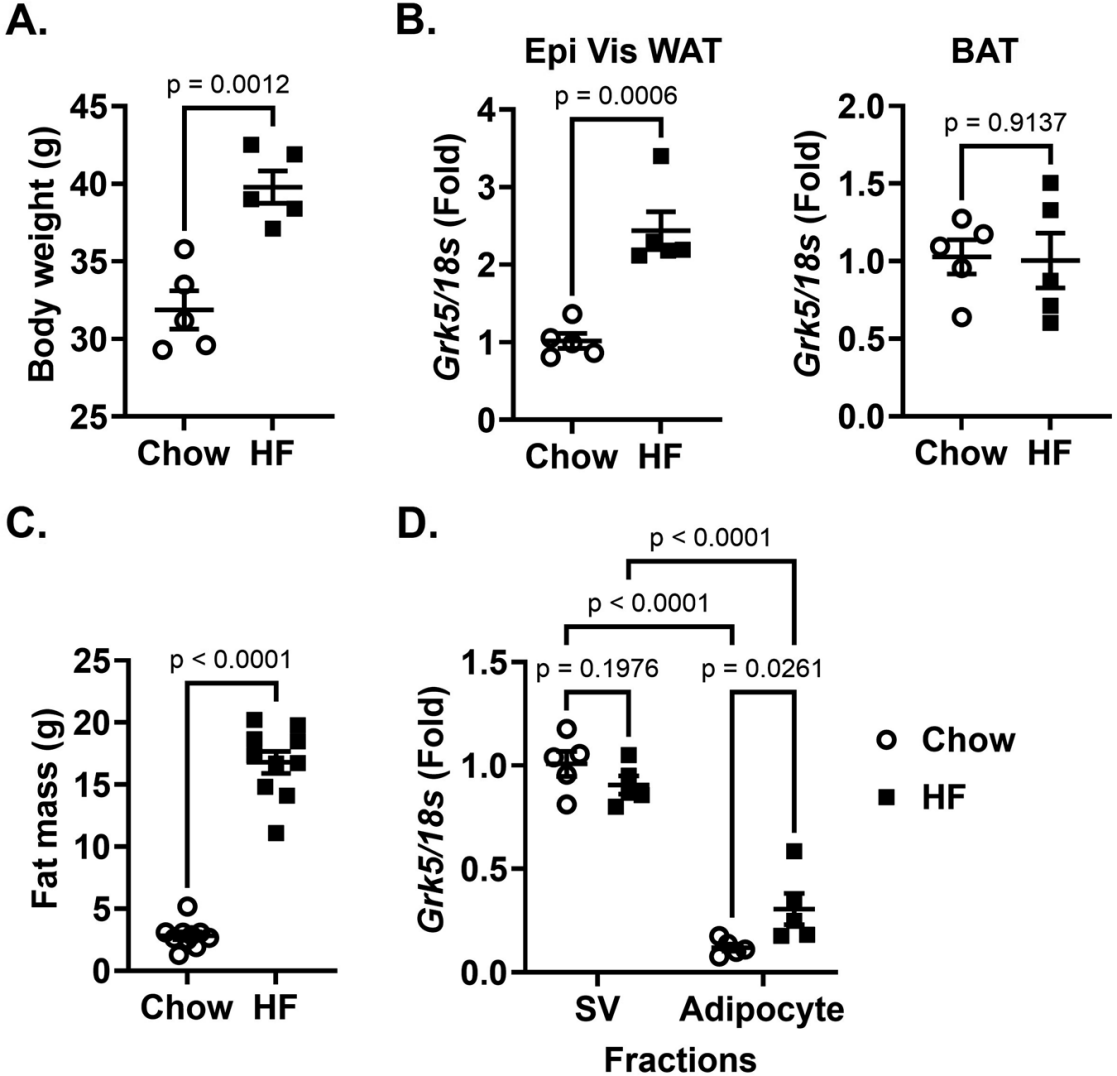
The relationship between *Grk5* expression and adiposity. **A** Eight-week-old male C57BL/6J mice were fed chow or a high fat diet (Envigo #TD 88137, 42% from fat, 0.2% total cholesterol) for 16 weeks and their body weight was measured (*n* = 5/diet group). **B** Mice were then fasted for 24 h and their epididymal (Epi) visceral (Vis) white adipose tissue (WAT) and brown adipose tissue (BAT) RNA was extracted and reverse-transcribed into cDNA for real-time PCR quantification of *Grk5* normalized to *18* *s* (endogenous control). **C** Six-week-old male C57BL/6J mice were fed chow or a high fat diet (Research Diets Inc #D12492, 60% from fat) for 12 weeks and their body composition such as fat mass was quantified by EcoMRI (*n* = 10/diet group). **D** Adipocyte fraction and stromal vascular (SV) cell fraction were isolated from the Epi Vis WAT of overnight fasted mice. Both fractions’ RNA was extracted and reverse-transcribed into cDNA for real-time PCR quantification of *Grk5* normalized to *18* *s* (endogenous control). All results are mean ± SEM, presented as the fold change compared to chow-fed mouse group and analyzed using a two-tailed Student’s unpaired *t* test (**A**–**C**), or the fold change compared to chow SV fractions and analyzed using a one-way ANOVA with Sidak multiple comparisons (**D**).

**Fig. 2:**
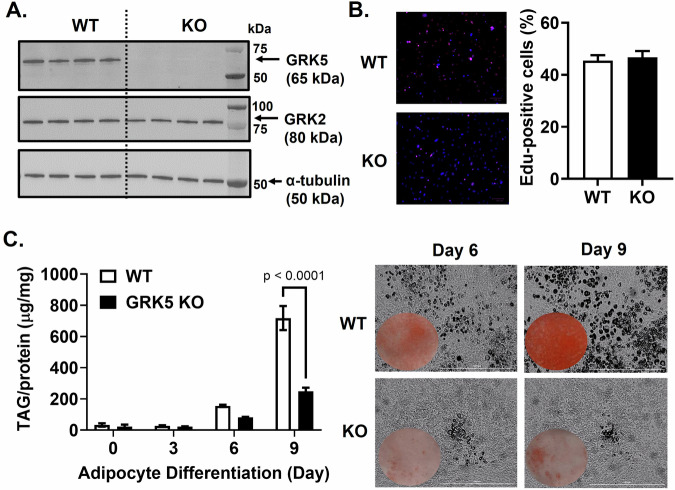
The effect of GRK5 deficiency on adipocyte differentiation. **A** Cellular proteins of undifferentiated wildtype (WT) control and GRK5 knockout (KO) 3T3-L1 preadipocytes (*n* = 4/genotype) were harvested and subjected to Western blot using anti-GRK5, anti-GRK2, and anti-α-tubulin antibodies. **B** After 2 days of growth, proliferation was assessed in undifferentiated WT and GRK5 KO 3T3-L1 preadipocytes (*n* = 6/genotype). The percentage of EdU-positive cells (pink) was calculated by merging EdU (red) and Hoechst 3342 (blue) staining. **C** WT and GRK5 KO 3T3-L1 cells (*n* = 3/genotype) were proliferated for 2 days (Day 0) and then differentiated into adipocytes for 9 days. Day 0, 3, 6, and 9 cells were lipid-extracted to measure triacylglycerol (TAG) mass by a colorimetric assay. Daily Cytation images at 10x magnification were taken during 9 days of adipocyte differentiation. All results are mean ± SEM and analyzed using a two-tailed Student’s unpaired *t* test (**B**) and a two-way ANOVA with Sidak multiple comparisons (**C**).

In support of previous work showing a positive correlation between *Grk5/GRK5* gene expression and adipose tissue mass in rats [4] and humans [6], we demonstrate here that high fat diet-induced obesity in male mice versus chow-fed lean male mice (Fig. 1A) displayed ~2-fold increased *Grk5* mRNA levels in epidydimal visceral white adipose tissue, but not in brown fat (Fig. 1B). The Grk5 mRNA levels in epidydimal visceral white adipose tissue, however, did not correspond to its protein levels (Supplementary Fig. 1A). Similar results were found in female mice fed chow or a high fat diet (Supplementary Fig. 1B). We further found that *Grk5* mRNA is more abundant in stromal vascular (SV) fractions than in adipocyte fractions isolated from the epidydimal visceral white adipose tissues of both chow-fed lean and a high fat diet-induced obesity in mouse (Fig. 1C, D). *Grk5* mRNA, however, did not increase in response to HFD in the SV fraction. The SV fractions contain stem cells, adipose progenitor cells (committed preadipocytes), endothelial cells, and immune cells. Thus, a GRK5 KO 3T3-L1 preadipocyte cell line was generated using CRISPR-Cas9 gene editing (Supplementary Fig. 2A) to investigate GRK5 function in adipocyte differentiation. The GRK5 protein deficiency in the KO cell line was confirmed by Western blots without affecting other GRK isoforms such as GRK2 expression (Fig. 2A). GRK3, GRK4, and GRK6 expressions were undetectable in the 3T3-L1 cell line (Data not shown). Because GRK1 and GRK7 belong to the retinal-specific GRK family [27, 28], we did not examine their expression in 3T3-L1 cells.

### GRK5 deficiency impairs adipocyte differentiation

**Fig. 3:**
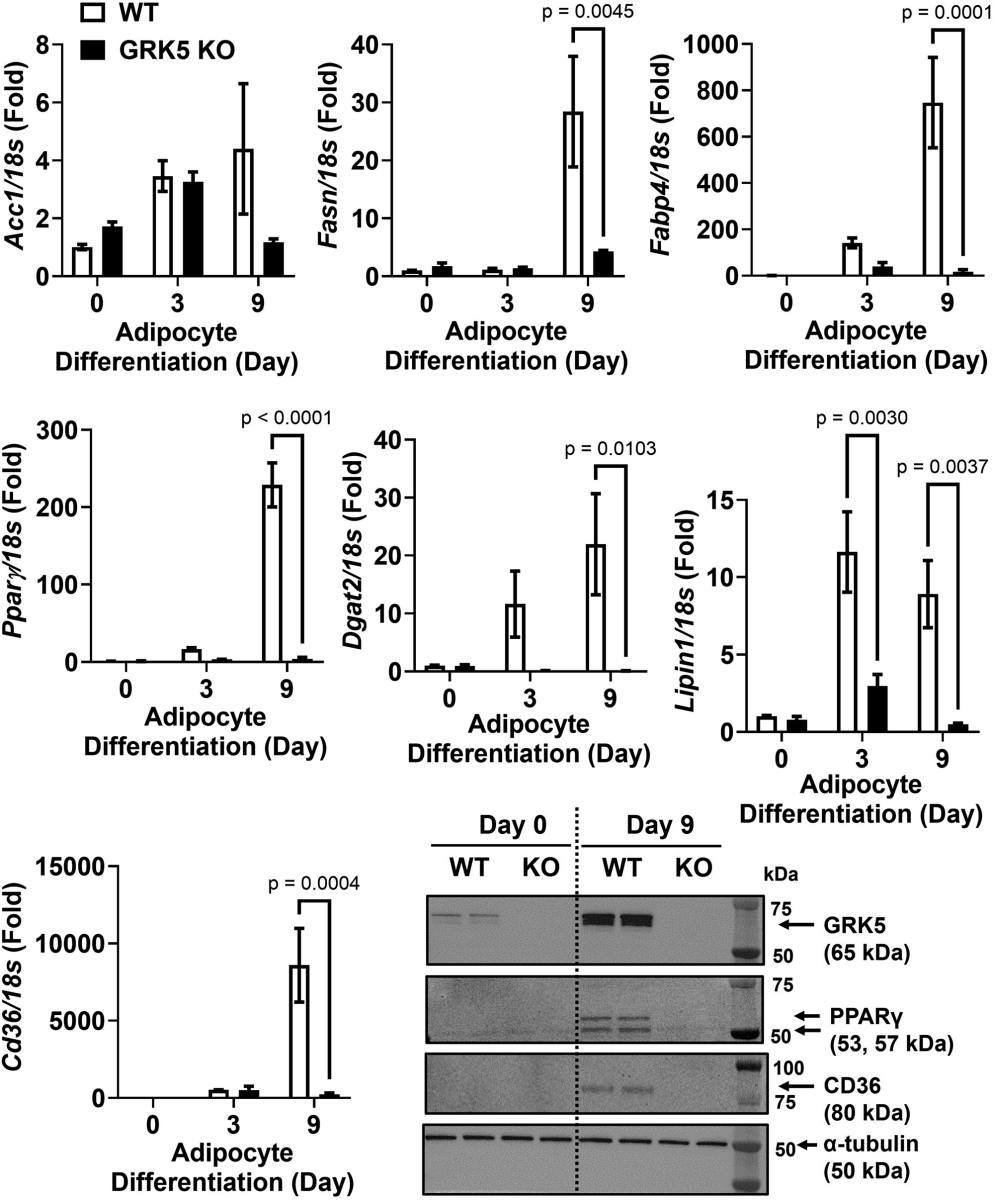
The effect of GRK5 deficiency on adipogenic and lipogenic gene and protein expression. Cellular RNA was extracted from wildtype (WT) control and GRK5 knockout (KO) 3T3-L1 cells (*n* = 3/genotype) and reverse-transcribed into cDNA for real-time PCR quantification of *Acc1, Pparγ*, *Fasn*, *Cd36, Fabp4*, *Dgat2*, and *Lipin1* normalized to *18* *s* (endogenous control). Cellular proteins of undifferentiated (Day 0) and differentiated (Day 9) WT control and GRK5 KO 3T3-L1 cells (*n* = 2/genotype) were harvested and subjected to Western blot using anti-GRK5, anti-PPARγ, anti-CD36, and anti-α-tubulin antibodies. All results are mean ± SEM and presented as the fold change compared to WT at Day 0 and analyzed using a two-way ANOVA with Sidak multiple comparisons.

First, we showed that WT and GRK5 KO cells proliferated similarly (Fig. 2B). We then found that, compared to WT, GRK5 KO cells had decreased accumulation of TAG and delayed development into mature adipocytes when exposed to adipogenic stimuli (Fig. 2C). We then examined several key genes that play a role in adipogenesis and lipid metabolism. Except *Acc1*, the mRNA levels of *Pparγ*, *Fasn*, *Fabp4*, *Cd36, Dgat2*, and *Lipin1* were decreased in GRK5 KO compared to WT adipocytes during Day 3-9 of differentiation (Fig. 3). The protein expression of CD36 and PPARγ was also decreased in Day 9 differentiated GRK5 KO compared to WT adipocytes (Fig. 3). These data suggest that GRK5 deletion in 3T3-L1 cells suppresses adipogenesis and lipid accumulation.

### GRK5 deficiency downregulates IGF-1 signaling and decreases ERK activation

**Fig. 4:**
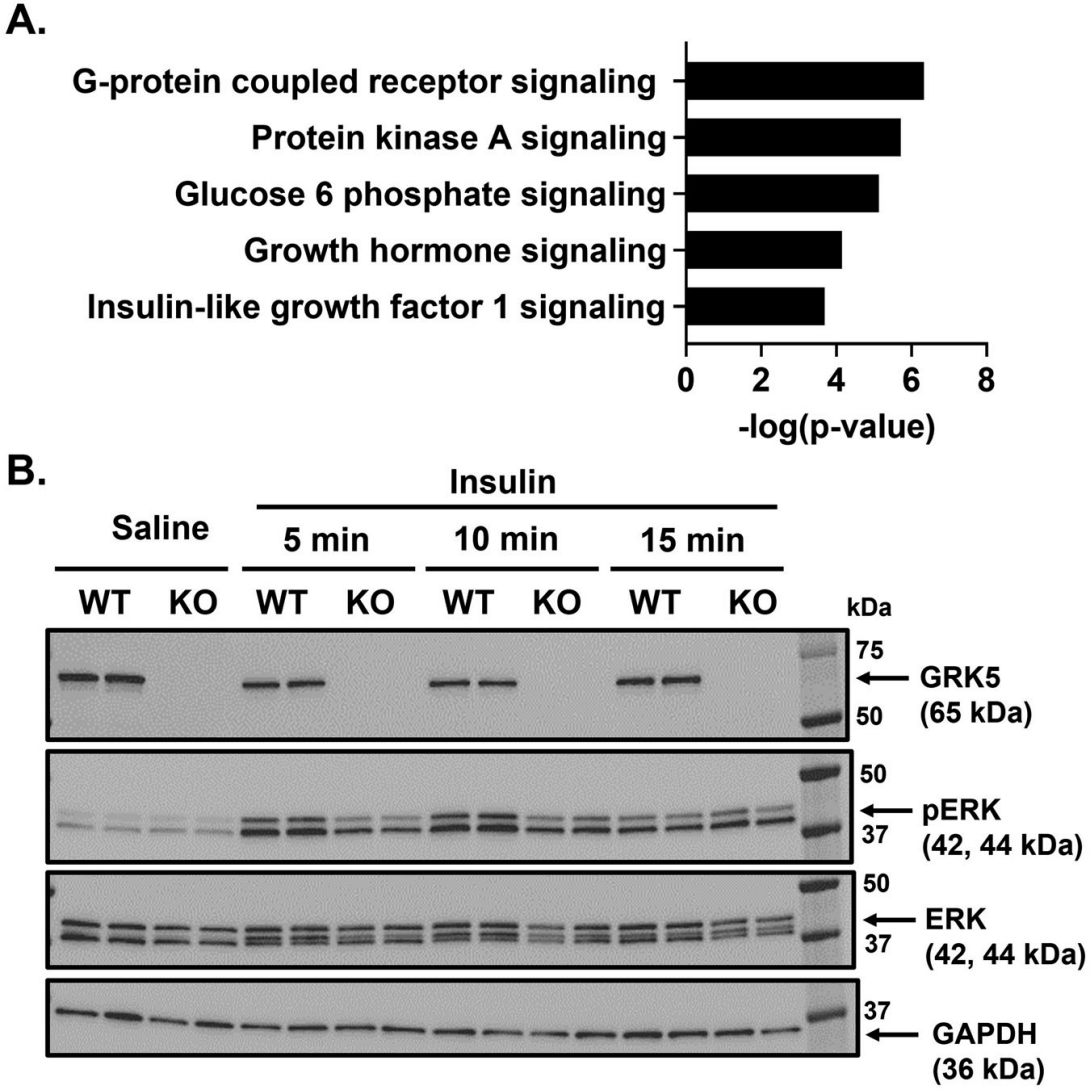
The potential underlying mechanisms of GRK5 in adipocyte differentiation. **A** Pathway analysis of RNA sequencing data using Limma. **B** Wildtype (WT) control and GRK5 knockout (KO) 3T3-L1 preadipocytes (*n* = 2/genotype) were differentiated for 2 days and treated with 1 μg/ml of insulin for 5, 10, and 15 min. Then, the cellular proteins were harvested and subjected to Western blot using anti-GRK5, anti-phosphorylated (p)-ERK, anti-ERK, and anti-GAPDH antibodies.

Next, we applied an unbiased RNA sequencing approach to identify differences in gene signatures between GRK5 KO and WT 3T3-L1 preadipocytes. We revealed 164 upregulated and 397 downregulated genes in GRK5 KO relative to WT preadipocyte cultures. Based on IPA pathway analysis datasets (Supplementary Table 1), we summarized the top 5 most dysregulated pathways involved in adipocyte differentiation (Fig. 4A). As expected, GPCR signaling (*p* = 4.677 × 10^−7^) and protein kinase A (PKA) signaling (*p* = 1.9498 × 10^−6^) were the top two significantly dysregulated pathways (Fig. 4A). GRK5 KO cells also have significantly dysregulated IGF-1 signaling (*p* = 2.04 × 10^−4^). GRKs are known signaling molecules shared between IGF-1R, a receptor tyrosine kinase, and GPCRs [29, 30].

Since ERK is an important downstream target for adipogenic insulin-activated IGF-1 receptor signaling for initiating adipogenesis [18, 19], we examined whether ERK phosphorylation was altered in GRK5 KO versus WT 3T3-L1 cell cultures treated with or without insulin. We found that GRK5 deletion in 3T3-L1 preadipocytes resulted in decreased ERK phosphorylation when compared to WT cells after insulin stimulation for 5 min (Fig. 4B**;** Quantification data in Supplementary Fig. 2B, C), indicating that GRK5 may regulate adipocyte differentiation through insulin/IGF-1 receptor and ERK pathways.

### GRK5 inhibitor reduces adipocyte differentiation

**Fig. 5:**
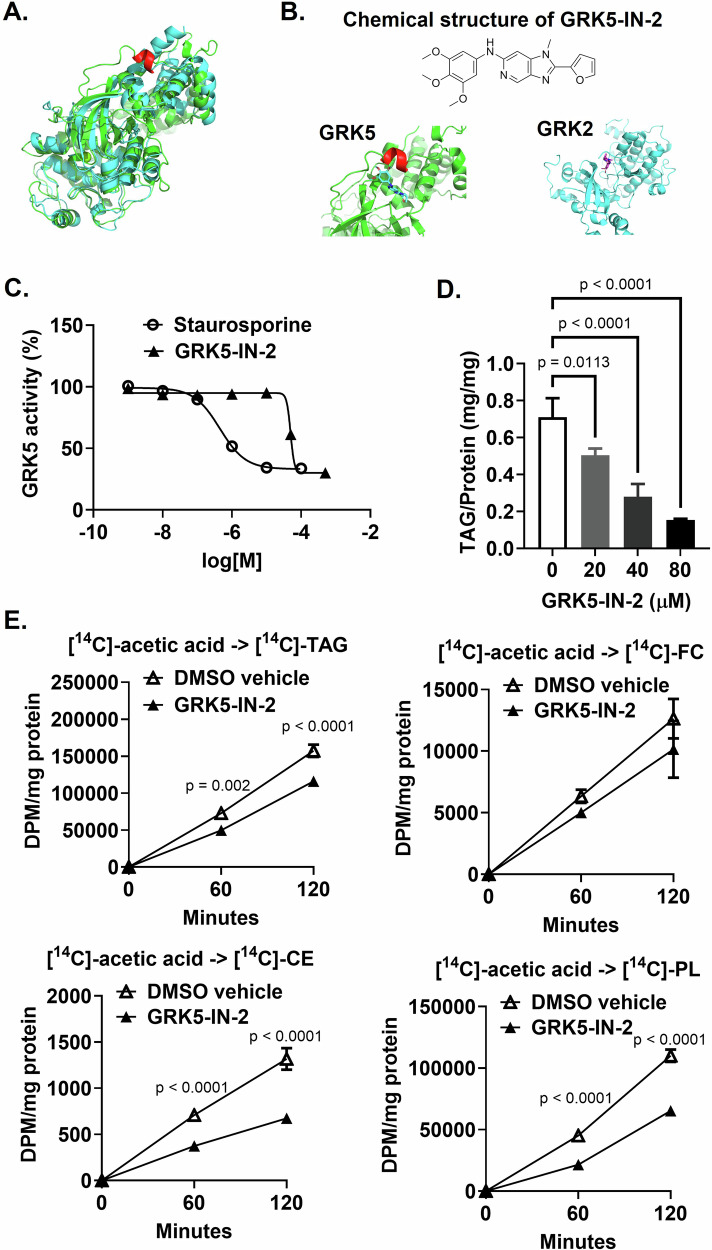
The effect of a GRK5 inhibitor on adipocyte differentiation. **A** Alignment of the GRK5 (green) and GRK2 (cyan) crystal structures. There is an alpha helix (shown in red) present near the binding pocket in GRK5, not present in GRK2. **B** Relative docking positions of GRK5-IN-2 in GRK5 and GRK2. **C** The dose-response curves of GRK5-IN-2 and staurosporine were determined by a GRK5 kinase system and a luminescent ADP detection assay. **D** Wildtype 3T3-L1 preadipocytes were differentiated and concurrently treated without (DMSO vehicle) or with GRK5-IN-2 (*n* = 3/dose) for 7 days. Cells were lipid-extracted to measure triacylglycerol (TAG) mass by an enzymatic colorimetric assay. **E** Day 3 differentiated wildtype 3T3-L1 cell cultures were pretreated without (DMSO vehicle) or with GRK5-IN-2 (40 μM) for 30 min and then treated with 1 μg/ml of insulin plus 0.5 μCi/ml of [1,2-^14^C]-acetic acid for 60 and 120 min (*n* = 3/time point). Cells were lipid-extracted, and TAG, free cholesterol (FC), cholesteryl ester (CE), and phospholipid (PL) were separated using thin layer chromatography. [^14^C]-TAG, [^14^C]-FC, [^14^C]-CE, and [^14^C]-PL were quantified by liquid scintillation counting. All results are mean ± SEM and analyzed using a two-way ANOVA with Sidak multiple comparisons.

Because genetic deletion of GRK5 is not a viable therapeutic option, we identified a small molecule GRK5 inhibitor, GRK5-IN-2, and assessed its effect on adipocyte differentiation. The 3T3-L1 cells expressed both GRK2 and GRK5 (Fig. 2A), so we first examined whether GRK5-IN-2 inhibits GRK2 or GRK5 more efficiently by using predictive computational molecular modeling. GRK2 and GRK5 protein structures were aligned to determine their root mean square deviation (RMSD), a value commonly used to measure the similarly between two protein structures, where a value less than 1 Å indicates greater structural similarities. The RMSD value for GRK5 and GRK2 alignment is 1.668 Å (Fig. 5A), demonstrating that the GRK5 and GRK2 proteins have different structures. Indeed, there is an additional α-helix in GRK5 adjacent to the predicted binding site for GRK5-IN-2 altering the binding modes of the inhibitor (Fig. 5B). Thus, the affinity of the binding mode for GRK5-IN-2 to GRK5 was −8.0 kcal/mol and to GRK2 was −6.6 kcal/mol suggesting that GRK5-IN-2 clearly has an inhibitory preference for GRK5 over GRK2.

Next, using a GRK5 kinase system and a luminescent ADP detection assay, we found that GRK5-IN-2 had a half maximal inhibitory concentration (IC50) of 49.7 µM as compared to staurosporine, the reference compound with an IC50 of 0.4 µM (Fig. 5C). We found that GRK5-IN-2 treatment significantly decreased TAG synthesis during 7 days of WT 3T3-L1 adipocyte differentiation in a dose-dependent manner (Fig. 5D). Next, we performed functional characterization. Day 3 differentiated WT cell cultures were treated with GRK5-IN-2 and labeled with [^14^C]-acetic acid to determine de novo lipogenesis or [^3^H]-oleic acid to measure fatty acid uptake and esterification. GRK5-IN-2 inhibitor treatment significantly decreased the rate of TAG, cholesteryl ester (CE), and phospholipid (PL), but not free cholesterol (FC), synthesis from [^14^C]-acetic acid (Fig. 5E). However, GRK5 inhibition did not affect [^3^H]-oleic acid uptake as well as [^3^H]-TAG, [^3^H]-CE and [^3^H]-PL formation from [^3^H]-oleic acid (Supplementary Fig. 3). These data suggest that the effect of GRK5 inhibition by GRK5-IN-2 is on insulin-stimulated de novo lipogenesis, not on fatty acid uptake and esterification into lipids.

## Discussion

In the current study we show that *Grk5* is expressed in the stromal vascular fraction of white adipose tissue in mice, and that GRK5 KO preadipocytes fail to differentiate into mature adipocytes after 9 days of differentiation, possibly as a result of impaired insulin-stimulated IGF-1 receptor and ERK activation. We further demonstrate that a novel GRK5 inhibitor, GRK5-IN-2, leads to decreased insulin-stimulated de novo TAG biosynthesis. Together, these results emphasize the importance of GRK5 in adipocyte differentiation, suggest a mechanistic pathway for its action, and identify a novel inhibitor that may have therapeutic promise. These important mechanistic in vitro studies support previous in vivo studies implicating *Grk5* as a causal gene for obesity.

The current work supports previous findings by Wang et al. [5] showing that diet-induced obesity in mice versus chow-fed lean mice had increased GRK5 mRNA levels (~2 fold) in white, but not in brown adipose tissue (Fig. 1A, B).We also show here that *Grk5* mRNA is abundant in the stromal vascular fraction, but not adipocyte fraction, of mouse white adipose tissue (Fig. 1C), supporting previous findings [10–12]. Interestingly, there were no differences in GRK5 protein levels in response to HFD in whole tissue, nor were there differences in *Grk5* mRNA in response to HFD in the SV fraction. These findings suggest that the role of *Grk5* in adipose tissue differentiation and/or obesity is not dependent on a HFD and further implicate *Grk5* as a causal, as opposed to reactive, obesity gene. This is not surprising given that our genetic mapping studies were conducted in outbred rats on normal chow [4]. In an effort to validate GRK5 as an important adiposity gene, as well as study the mechanistic role of GRK5 in adipose differentiation in vitro, we created a novel GRK5 KO 3T3-L1 preadipocyte model. One limitation of this study was that we only examined GRK5’s loss of function effect on adipogenesis, rather than its gain of function/overexpression effect.

During adipogenic stimuli, GRK5 KO 3T3-L1 preadipocytes acquired adipocyte morphology slower and accumulated less TAG than WT control cells (Fig. 2), potentially by suppressing expression of several adipogenic and lipogenic genes (Fig. 3). In support of our data, Wang et al. observed that adipogenic gene expression (e.g., *Pparγ*, *Fasn*, and *Fabp4*) is decreased in white adipose tissue of whole body GRK5 KO mice compared to their littermate control mice on a high fat diet [5]. However, the mechanisms by which GRK5 regulates adipogenesis in preadipocytes are unknown. Therefore, we performed RNA sequencing and pathway analysis and found that IGF-1 signaling may be a potential underlying mechanism (Fig. 4A).

Canonically, GRKs terminate GPCR signaling though phosphorylation of cognate receptors, resulting in recruitment of β-arrestin, which then facilitates receptor internalization and degradation [7]. As a receptor tyrosine kinase, IGF-1 receptor has recently been found to “borrow” molecular components from GPCR signaling, such as GRKs and β-arrestins [29, 30]. For example, Zheng et al. reported that GRK2 and GRK6 can physically bind to IGF-1 receptor and phosphorylate IGF-1 receptor at serines 1248 and 1291. These serine phosphorylated binding sites on IGF-1 receptor promoted β-arrestin1 recruitment [31]. However, there was an opposing effect of GRK2 and GRK6: knockdown of GRK2 increased whereas knockdown of GRK6 decreased IGF-1 receptor degradation and β-arrestin1-mediated ERK activation [31]. Because GRK5 and GRK6 belong to the same subfamily [7], we hypothesized and demonstrated that in a similar manner to GRK6 knockdown, GRK5 deficiency reduced ERK activation in insulin-treated 3T3-L1 preadipocyte cultures (Fig. 4B). Preadipocytes express primarily IGF-1 receptors rather than insulin receptors, so insulin in an adipogenic cocktail promotes adipocyte differentiation by activating IGF-1 receptor and its downstream ERK pathways [19, 32]. Future studies are needed to investigate whether GRK5 can bind and phosphorylate insulin-stimulated IGF-1 receptor which will then activate ERK pathway in preadipocytes to promote adipogenesis.

Since genetic deletion is not a viable therapeutic option, we have identified GRK5-IN-2, a pyridine-based bicyclic compound [33], as a commercially available small molecule GRK5 inhibitor. Our cell data demonstrated that GRK5-IN-2 treatment resulted in decreased and/or delayed adipogenesis much like our GRK5 KO cell line, as well as decreased de novo lipogenesis (Fig. 5), suggesting that GRK5 inhibition may be a therapeutic target for treating and preventing obesity. Although there are other GRK5 inhibitors such as KR-39038 [34], we chose GRK5-IN-2 because synthesizing KR-39038 is very time-consuming and expensive, which may limit its use in clinical settings. The above studies support previous work [4, 5], suggesting an important role for GRK5 in adipocyte differentiation.

These mechanistic studies indicate that GRK5 is acting through the IGF-1 receptor and ERK pathway, although future studies are needed to demonstrate a direct link between these proteins. In order to study mechanism, all current studies were done in vitro, and future studies will be required to test the role of GRK5-IN-2 in vivo. Toward that goal, we are currently conducting preclinical studies using GRK5-IN-2 to see if we can replicate our cell results in a mouse model of diet-induced obesity and to determine whether GRK5 inhibition could be a valuable translation target.

## Supplementary information

Supplemental Figures 1-3

Supplemental Table 1

## Data Availability

All the data generated during and/or analyzed during the current study are available from the corresponding author on reasonable request.

## References

[1] Liu B, Du Y, Wu Y, Snetselaar LG, Wallace RB, Bao W. Trends in obesity and adiposity measures by race or ethnicity among adults in the United States 2011-18: population based study. BMJ. 2021;372:n365. 33727242 10.1136/bmj.n365 PMC7961695

[2] Ward ZJ, Bleich SN, Cradock AL, Barrett JL, Giles CM, Flax C, et al. Projected U.S. State-Level Prevalence of Adult Obesity and Severe Obesity. N Engl J Med. 2019;381:2440–50. 31851800 10.1056/NEJMsa1909301

[3] Schelbert KB. Comorbidities of obesity. Prim Care. 2009;36:271–85. 19501243 10.1016/j.pop.2009.01.009

[4] Hong-Le T, Crouse WL, Keele GR, Holl K, Seshie O, Tschannen M, et al. Genetic Mapping of Multiple Traits Identifies Novel Genes for Adiposity, Lipids, and Insulin Secretory Capacity in Outbred Rats. Diabetes. 2023;72:135–48. 36219827 10.2337/db22-0252 PMC9797320

[5] Wang F, Wang L, Shen M, Ma L. GRK5 deficiency decreases diet-induced obesity and adipogenesis. Biochem Biophys Res Commun. 2012;421:312–7. 22507984 10.1016/j.bbrc.2012.04.006

[6] Sharma NK, Sajuthi SP, Chou JW, Calles-Escandon J, Demons J, Rogers S, et al. Tissue-Specific and Genetic Regulation of Insulin Sensitivity-Associated Transcripts in African Americans. J Clin Endocrinol Metab. 2016;101:1455–68. 26789776 10.1210/jc.2015-3336 PMC4880154

[7] Komolov KE, Benovic JL. G protein-coupled receptor kinases: Past, present and future. Cell Signal. 2018;41:17–24. 28711719 10.1016/j.cellsig.2017.07.004 PMC5722692

[8] GTExPortal. GRK5. Available from: https://www.gtexportal.org/home/gene/GRK5#gtexmenu.

[9] NCBI. Grk5 G protein-coupled receptor kinase 5 [Mus musculus (house mouse). 2024. Available from: https://www.ncbi.nlm.nih.gov/gene/14773.

[10] Atlas THP. GRK5. Available from: https://www.proteinatlas.org/ENSG00000198873-GRK5/tissue+cell+type.

[11] Ferrero R, Rainer P, Deplancke B. Toward a Consensus View of Mammalian Adipocyte Stem and Progenitor Cell Heterogeneity. Trends Cell Biol. 2020;30:937–50. 33148396 10.1016/j.tcb.2020.09.007

[12] Emont MP, Jacobs C, Essene AL, Pant D, Tenen D, Colleluori G, et al. A single-cell atlas of human and mouse white adipose tissue. Nature. 2022;603:926–33. 35296864 10.1038/s41586-022-04518-2 PMC9504827

[13] Ikoma-Seki K, Nakamura K, Morishita S, Ono T, Sugiyama K, Nishino H, et al. Role of LRP1 and ERK and cAMP Signaling Pathways in Lactoferrin-Induced Lipolysis in Mature Rat Adipocytes. PLoS One. 2015;10:e0141378. 26506094 10.1371/journal.pone.0141378 PMC4623961

[14] Petersen RK, Madsen L, Pedersen LM, Hallenborg P, Hagland H, Viste K, et al. Cyclic AMP (cAMP)-mediated stimulation of adipocyte differentiation requires the synergistic action of Epac- and cAMP-dependent protein kinase-dependent processes. Mol Cell Biol. 2008;28:3804–16. 18391018 10.1128/MCB.00709-07 PMC2423297

[15] Reusch JE, Colton LA, Klemm DJ. CREB activation induces adipogenesis in 3T3-L1 cells. Mol Cell Biol. 2000;20:1008–20. 10629058 10.1128/mcb.20.3.1008-1020.2000 PMC85218

[16] Yeh WC, Cao Z, Classon M, McKnight SL. Cascade regulation of terminal adipocyte differentiation by three members of the C/EBP family of leucine zipper proteins. Genes Dev. 1995;9:168–81. 7531665 10.1101/gad.9.2.168

[17] Wu Z, Bucher NL, Farmer SR. Induction of peroxisome proliferator-activated receptor gamma during the conversion of 3T3 fibroblasts into adipocytes is mediated by C/EBPbeta, C/EBPdelta, and glucocorticoids. Mol Cell Biol. 1996;16:4128–36. 8754811 10.1128/mcb.16.8.4128 PMC231409

[18] Smith PJ, Wise LS, Berkowitz R, Wan C, Rubin CS. Insulin-like growth factor-I is an essential regulator of the differentiation of 3T3-L1 adipocytes. J Biol Chem. 1988;263:9402–8. 2967822

[19] Prusty D, Park BH, Davis KE, Farmer SR. Activation of MEK/ERK signaling promotes adipogenesis by enhancing peroxisome proliferator-activated receptor gamma (PPARgamma) and C/EBPalpha gene expression during the differentiation of 3T3-L1 preadipocytes. J Biol Chem. 2002;277:46226–32. 12270934 10.1074/jbc.M207776200

[20] Seramur ME, Sink S, Cox AO, Furdui CM, Key CC. ABHD4 regulates adipocyte differentiation in vitro but does not affect adipose tissue lipid metabolism in mice. J Lipid Res. 2023;64:100405. 37352974 10.1016/j.jlr.2023.100405 PMC10400869

[21] Bolger AM, Lohse M, Usadel B. Trimmomatic: a flexible trimmer for Illumina sequence data. Bioinformatics. 2014;30:2114–20. 24695404 10.1093/bioinformatics/btu170 PMC4103590

[22] Dobin A, Davis CA, Schlesinger F, Drenkow J, Zaleski C, Jha S, et al. STAR: ultrafast universal RNA-seq aligner. Bioinformatics. 2013;29:15–21. 23104886 10.1093/bioinformatics/bts635 PMC3530905

[23] Liao Y, Smyth GK, Shi W. featureCounts: an efficient general purpose program for assigning sequence reads to genomic features. Bioinformatics. 2014;30:923–30. 24227677 10.1093/bioinformatics/btt656

[24] Law CW, Chen Y, Shi W, Smyth GK. voom: Precision weights unlock linear model analysis tools for RNA-seq read counts. Genome Biol. 2014;15:R29. 24485249 10.1186/gb-2014-15-2-r29 PMC4053721

[25] Morris GM, Huey R, Lindstrom W, Sanner MF, Belew RK, Goodsell DS, et al. AutoDock4 and AutoDockTools4: Automated docking with selective receptor flexibility. J Comput Chem. 2009;30:2785–91. 19399780 10.1002/jcc.21256 PMC2760638

[26] O’Boyle NM, Banck M, James CA, Morley C, Vandermeersch T, Hutchison GR. Open Babel: An open chemical toolbox. J Cheminform. 2011;3:33. 21982300 10.1186/1758-2946-3-33 PMC3198950

[27] Zhao X, Huang J, Khani SC, Palczewski K. Molecular forms of human rhodopsin kinase (GRK1). J Biol Chem. 1998;273:5124–31. 9478965 10.1074/jbc.273.9.5124

[28] Chen CK, Zhang K, Church-Kopish J, Huang W, Zhang H, Chen YJ, et al. Characterization of human GRK7 as a potential cone opsin kinase. Mol Vis. 2001;7:305–13. 11754336

[29] Janssen J. New Insights from IGF-IR Stimulating Activity Analyses: Pathological Considerations. Cells. 2020;9:862. 32252327 10.3390/cells9040862 PMC7226833

[30] Hupfeld CJ, Olefsky JM. Regulation of receptor tyrosine kinase signaling by GRKs and beta-arrestins. Annu Rev Physiol. 2007;69:561–77. 17002595 10.1146/annurev.physiol.69.022405.154626

[31] Zheng H, Worrall C, Shen H, Issad T, Seregard S, Girnita A, et al. Selective recruitment of G protein-coupled receptor kinases (GRKs) controls signaling of the insulin-like growth factor 1 receptor. Proc Natl Acad Sci USA. 2012;109:7055–60. 22509025 10.1073/pnas.1118359109 PMC3345003

[32] Entingh-Pearsall A, Kahn CR. Differential roles of the insulin and insulin-like growth factor-I (IGF-I) receptors in response to insulin and IGF-I. J Biol Chem. 2004;279:38016–24. 15247278 10.1074/jbc.M313201200

[33] Ullrich A, Falcenberg M. inventors; Max-Planck-Gesellschaft zur Förderung, assignee. Imidazo[4,5-c]pyridine and pyrrolo[3,2-c]pyridine compounds as G-protein-coupled receptor kinase 5 (GRK5) modulators. Germany. 2014. https://patents.google.com/patent/EP2818472A1/en.

[34] Lee JH, Seo HW, Ryu JY, Lim CJ, Yi KY, Oh KS, et al. KR-39038, a Novel GRK5 Inhibitor, Attenuates Cardiac Hypertrophy and Improves Cardiac Function in Heart Failure. Biomol Ther. 2020;28:482–9. 10.4062/biomolther.2020.129 PMC7457178 32856617

