# Supplementary material for: GRK5 is required for adipocyte differentiation through ERK activation
Source: Int J Obes (Lond). 2025 Jan 21;49(5):855–63. doi: 10.1038/s41366-025-01712-w (PMC12095040; doi:10.1038/s41366-025-01712-w)
Supplement: Supplementary file 1 — Supplemental Figures 1-3 [file 41366_2025_1712_MOESM1_ESM.docx]

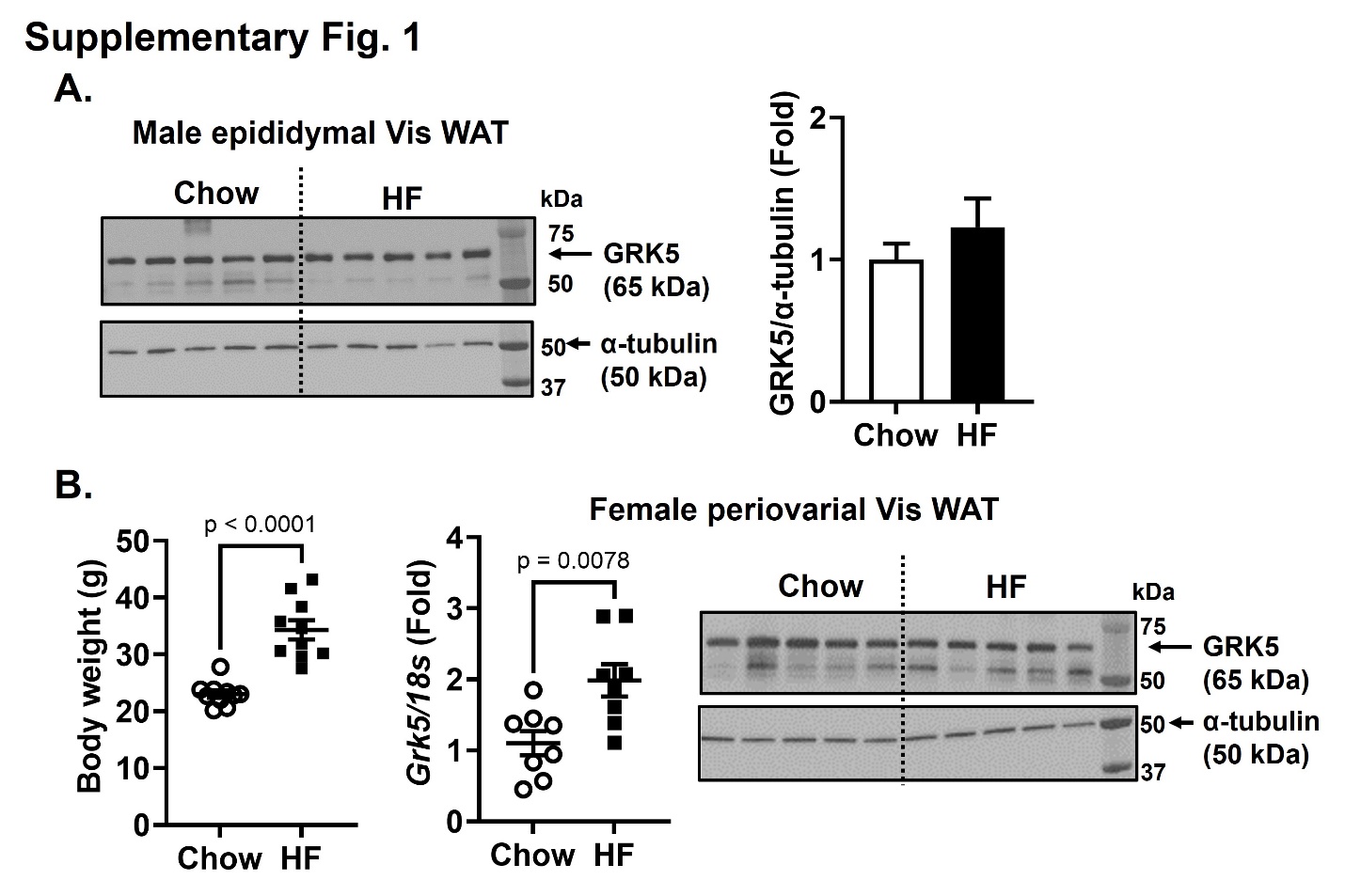


**Supplementary Fig. 1.** **(A)** Six-week-old male C57BL/6J mice were fed chow or a high fat diet (Research Diets Inc #D12451, 45% from fat) for 16 weeks. **(B)** Six-week-old female C57BL/6J mice were fed chow or a high fat diet (Research Diets Inc #D12492, 60% from fat) for 12 weeks. After overnight fasting, mice were weighed, and then male epididymal or female periovarial visceral (Vis) white adipose tissue (WAT) was collected for GRK5 gene and protein expression. All results are mean ± SEM, presented as the fold change compared to chow-fed mouse group and analyzed using a two-tailed Student’s unpaired t-test.


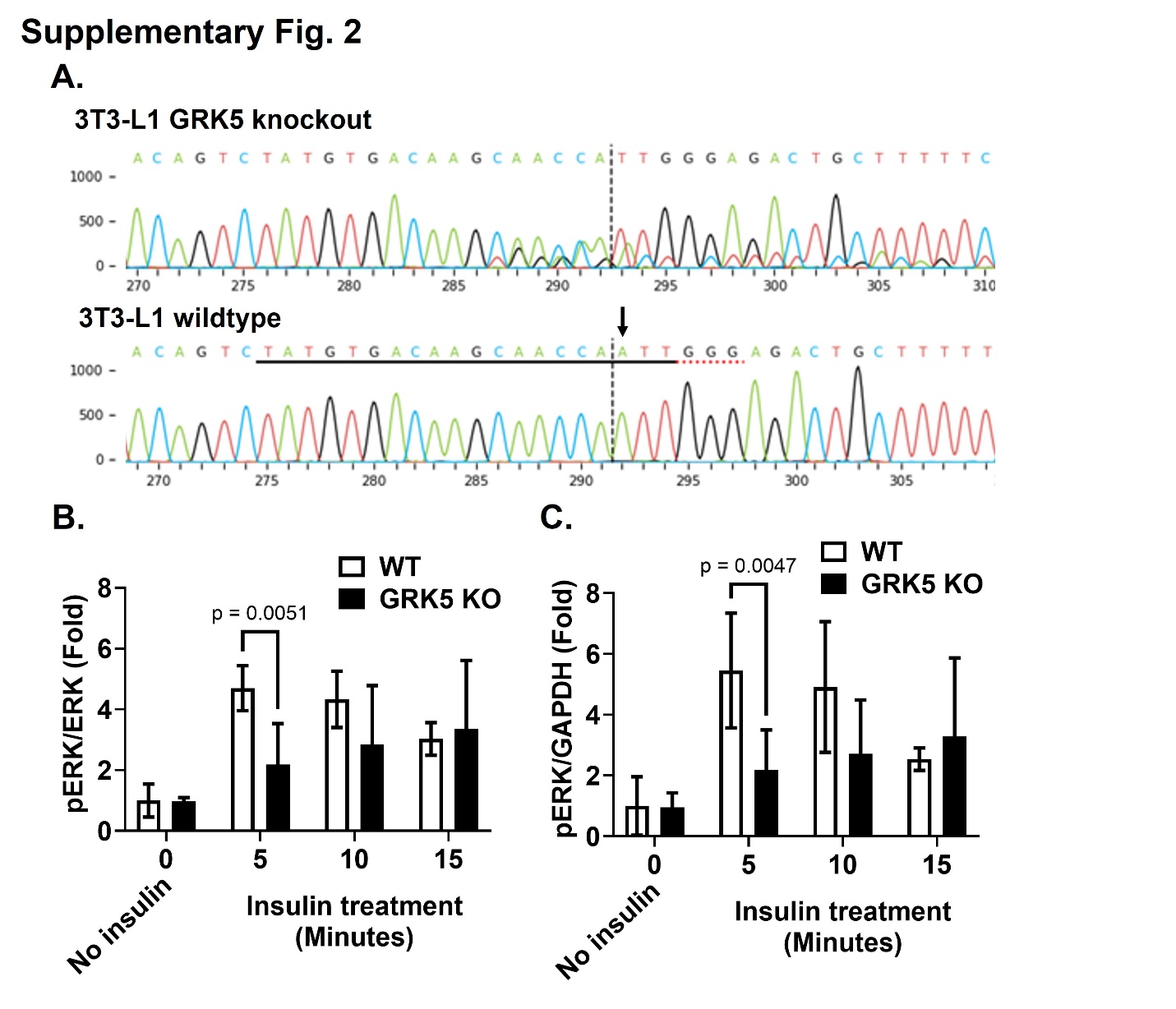


**Supplementary Fig. 2. (A)** The Sanger sequence view showing edited (knockout) and wildtype sequences of Grk5 in the region around the guide sequence. The horizontal black underlined region represents the guide sequence targeting exon 3 of Grk5. The horizontal red dotted underline is the PAM site. The vertical black dotted line represents the actual cut site. The knockout clone was cut and had a nucleotide removed compared to the wildtype clone (an arrow pointing down) during the non-homologous end joining repair process, resulting in a frameshift mutation that causes premature termination of translation at a new nonsense codon. **(B-C)** Quantification of pERK/ERK and pERK/GAPDH protein expression ratio for **Fig. 4B** (n=4/treatment).


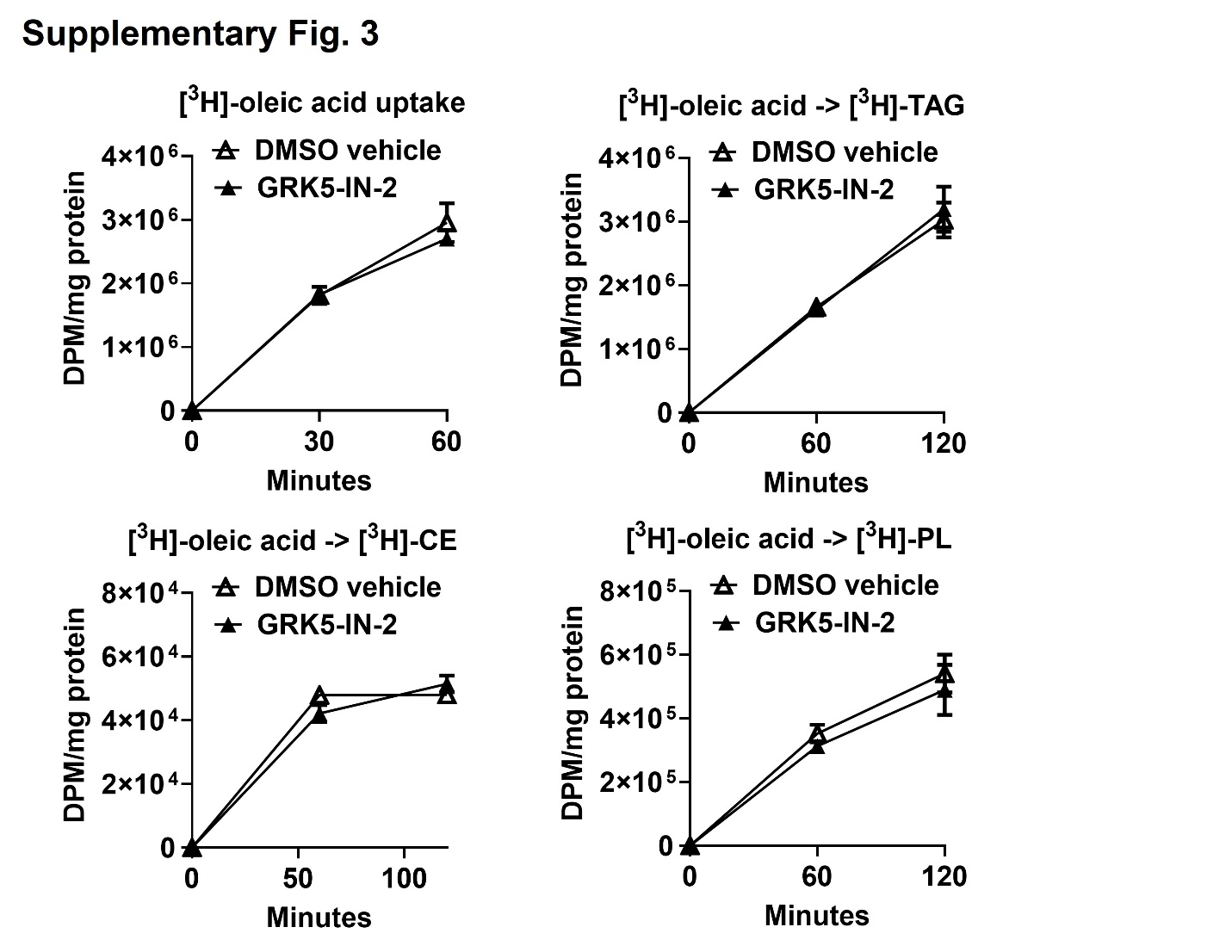


**Supplementary Fig. 3.** Day 3 differentiated 3T3-L1 pre-adipocyte cultures were pre-treated without or with GRK5-IN-2 for 30 minutes and then treated with insulin plus 5 μCi/ml of [9,10-^3^H(N)]-oleic acid for 60 and 120 minutes (n=3/time point). Cells were lipid-extracted, and triacylglycerol (TAG), cholesteryl ester (CE), and phospholipid (PL) were separated using thin layer chromatography. Cellular [^3^H], [^3^H]-TAG, [^3^H]-CE, and [^3^H]-PL were quantified by liquid scintillation counting. All results are mean ± SEM and analyzed using a two-way ANOVA with Sidak multiple comparisons.
